# Exposure of Endothelial Cells to Physiological Levels of Myeloperoxidase-Modified LDL Delays Pericellular Fibrinolysis

**DOI:** 10.1371/journal.pone.0038810

**Published:** 2012-06-19

**Authors:** Karim Zouaoui Boudjeltia, Jalil Daher, Pierre Van Antwerpen, Nicole Moguilevsky, Paul Delree, Jean Ducobu, Martine Raes, Bassam Badran, Michel Vanhaeverbeek, Dany Brohee, Claude Remacle, Luc Vanhamme

**Affiliations:** 1 Experimental Medicine Laboratory, Université Libre de Bruxelles 222 Unit, CHU Charleroi, A. Vésale, Montigny-Le-Tilleul, Belgium; 2 Laboratory of Molecular Parasitology, IBMM, Faculty of Science, Université Libre de Bruxelles, Gosselies, Belgium; 3 Laboratory of Pharmaceutical Chemistry, Faculty of Pharmacy, and Analytical Platform of the Faculty of Pharmacy, Université Libre de Bruxelles, Brussels, Belgium; 4 Technology Transfer Office, Faculté Universitaire Notre Dame de la Paix, Namur, Belgium; 5 Pathology and Genetic Institute, Gosselies, Belgium; 6 Laboratory of Biochemistry and Cellular Biology, Faculté Universitaire Notre Dame de la Paix, Namur, Belgium; 7 Department of Biochemistry, Laboratory of Immunology, Lebanese University, Faculty of Sciences, Hadath-Beirut, Lebanon; 8 Institute of life Sciences, Laboratory of Cellular Biology, Université Catholique de Louvain, Louvain-La-Neuve, Belgium; Idaho State University, United States of America

## Abstract

**Background:**

Blood fluidity is maintained by a delicate balance between coagulation and fibrinolysis. The endothelial cell surface is a key player in this equilibrium and cell surface disruptions can upset the balance. We investigated the role of pericellular myeloperoxidase oxidized LDLs (Mox-LDLs) in this balance.

**Methods and Results:**

We designed a technical device that enabled us to monitor fibrinolysis in real-time at the surface of an endothelial cell line (EA.hy926), and showed that Mox-LDL decreased pericellular fibrinolysis. There were no changes in fibrinolysis when EA.hy926 endothelial cells were exposed to native LDL (24 hours) at doses of 10, 50, 100 and up to 1250 µg/ml. However, treatment of EA.hy926 endothelial cells with 10 and 50 µg/ml of Mox-LDL (physiological serum concentrations) increased the lysis time by 15 and 13%, respectively (p<0.001), although this effect was not present at higher concentrations of 100 µg/ml. This effect was not correlated with any changes in PAI-1 or t-PA or PA Receptor (PAR) expression. No effect was observed at the surface of smooth muscle cells used as controls.

**Conclusion:**

Our data link the current favorite hypothesis that modified LDL has a causal role in atheroma plaque formation with an old suggestion that fibrin may also play a causal role. Our data help complete the paradigm of atherosclerosis: Modified LDL locally enhances fibrin deposition (present work); fibrin deposits enhance endothelial permeability; this effect allows subendothelial accumulation of lipid and foam cells.

## Introduction

Atherosclerosis is a clinical condition for which multiple genetic and environmental causal factors have been proposed. The atherosclerotic process involves thickening of the arterial wall; this thickening is related to the accumulation of foam cells, macrophages that have engulfed large amounts of modified LDL particles. These macrophages differentiate from monocytes that are recruited to the endothelium and activated to express leukocyte adhesion molecules [1], [2]. These adhesins are themselves also induced by modified LDLs (more abundant in patients with hypercholesterolemia), and it is, therefore, widely accepted that they are involved in atherogenesis [3]. Observations also suggest that myeloperoxidase (MPO), a protein secreted by activated phagocytes, is a major physiological player in generating modified/oxidized (lipo)proteins [4], [5] via the production of hypochlorous acid (HOCl) from H_2_O_2_ and chloride [6]. HOCl-modified LDLs (HOCl-LDLs) are present in human atherosclerotic lesions, where they are located both in vascular cells and in extracellular spaces [4]. Clinical studies have shown that patients with MPO-deficiency or low blood levels of MPO have reduced risk of cardiovascular disease [5], [7]. Two other studies reported that serum MPO levels could predict prognosis in patients with acute coronary syndromes or chest pain [8], [9]. The circulating form of MPO can bind to LDL [10] because of its highly cationic isoelectric point (pI >10).

Early circumstantial observations also correlated fibrin deposition with atheroma plaque formation. It has, therefore, been proposed that a decrease in plasma or pericellular fibrinolytic capacity may predispose to atherogenesis [11], [12]. Recent clinical studies indeed confirm a hypofibrinolytic state in atherosclerotic patients [13].

The endothelial cell plasma membrane is a place where coagulation and fibrinolysis are balanced in a continuous, dynamic equilibrium. Endothelial cells themselves feed this process by secreting coagulation and fibrinolysis factors. For example, endothelial cells secrete at least three fibrinolysis regulators: Tissue-plasminogen activator (t-PA), urokinase-plasminogen activator (u-PA) and plasminogen activator inhibitor-1 (PAI-1). They also express specific receptors, which bind fibrinolysis factors (such as u-PA, t-PA, t-PA-PAI-1 complex or plasminogen) and, thereby, modulate their activity [14]. Therefore, any interference with the endothelial cell surface or gene expression has possible implications for fibrinolysis and vice-versa. For example, physical forces or clinical conditions confer a prothrombotic environment on the endothelial membrane because they enhance fibrin generation [15]. Conversely, fibrin deposition on confluent endothelial cells disorganizes their regular cobblestone arrangement and increases the monolayer permeability [16], [17], [18]. Fibrin also induces endothelial cells to produce and release interleukin (IL)-8, a leukocyte chemotactic factor [19].

Because of technical limitations, the interplay between endothelial cells, oxidized LDLs and fibrinolysis has never been properly analyzed. Using an up-to-date technical device that allows real-time monitoring of fibrinolysis, we show a negative effect of MPO-modified LDLs (Mox-LDLs) on pericellular fibrinolysis. We propose a model involving increased fibrin levels as an early event in the progression of atheroma lesions.

## Results

### Fibrinolytic Process at the Cell Surface

**Figure 1:**
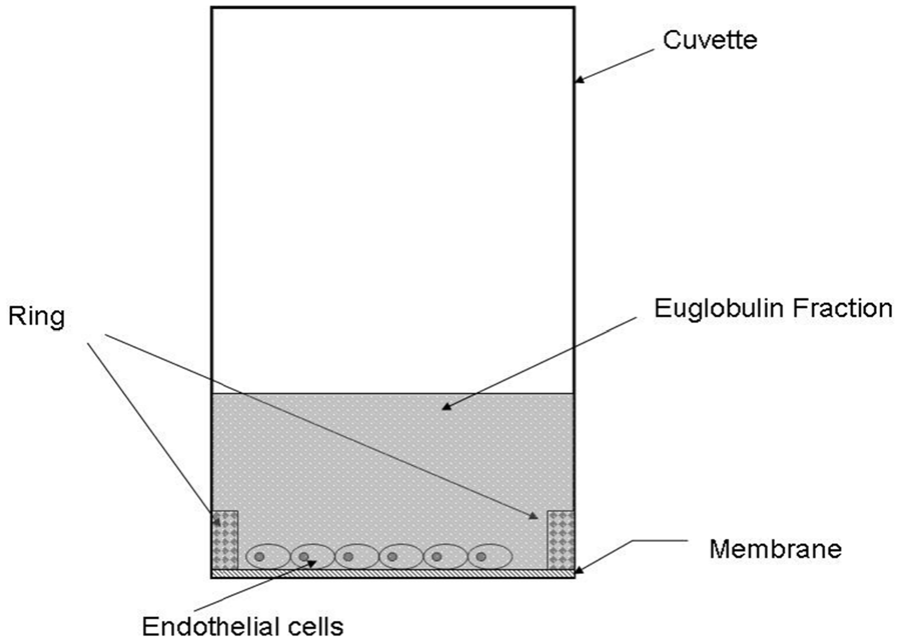
The experimental device. Fibrin formation and degradation occurred in adapted circular microcuvettes and were recorded by an automated device. To monitor the effect of cells on fibrinolysis taking place at their surface, cells were inoculated on collagen coated membranes; stuck at the bottom of glass circular micro-cuvettes; by a ring; and grown to confluence. The microcuvettes were inserted in the apparatus at 37°C, the euglobin fraction added and clot formation started by addition of thrombin. Coagulation and fibrinolysis were then monitored by the device. In control experiments, monitoring was performed in the presence of empty (cell-devoid) membranes or after addition of TNF-α to the cuvettes as described.

**Figure 2:**
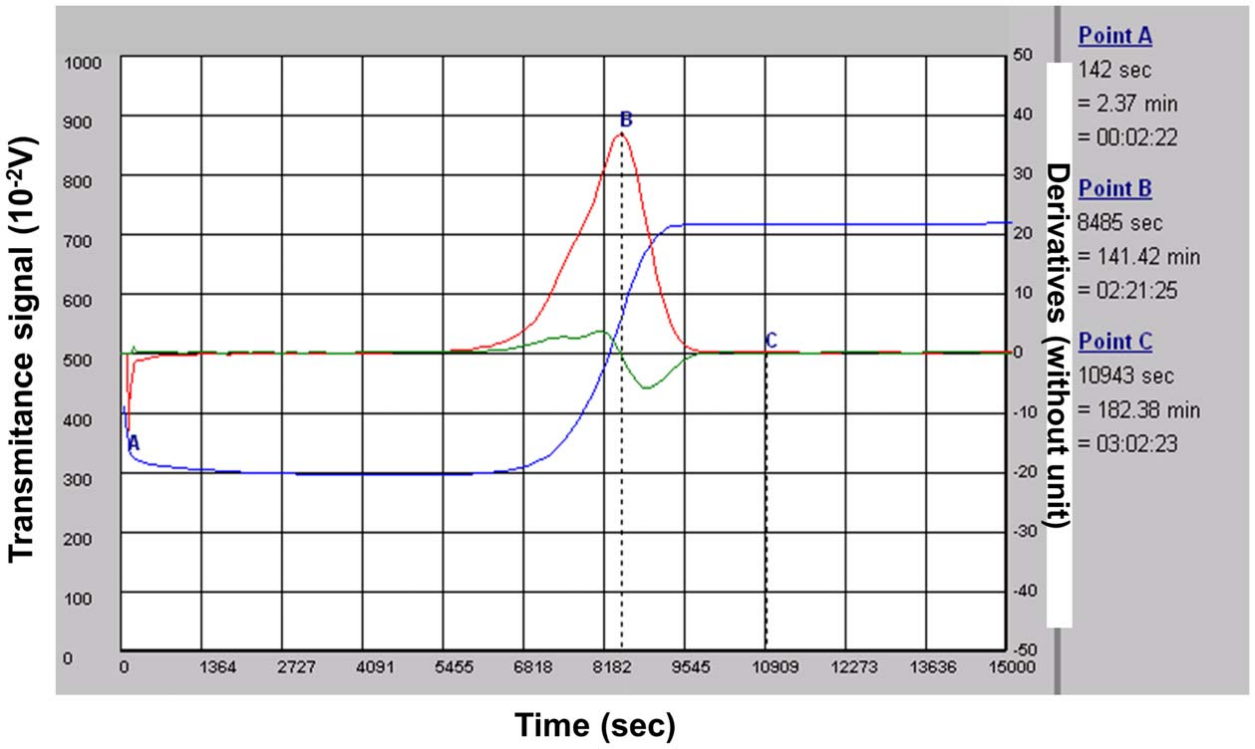
The course of the lysis process. A typical fibrinolysis monitoring**.** Euglobulin Clot Lysis Time (ECLT) is expressed in minutes. It is calculated from a clot lysis primary graph; this graph records the light transmittance at 680 nm as a function of time (measured in seconds): blue curve. Typically the transmittance decreases very quickly after thrombin addition which triggers clot formation and, after a latency time, fibrinolysis progressively restores transmittance. The ECLT is then determined from a mathematical analysis of the recorded lysis curve. Primary and secondary derivations of the recorded values generate the red and green graphs respectively. These graphs allow calculation of the B point, the peak to fibrin clot lysis, and the C point, the end of the complete fibrinolysis process.

**Figure 3:**
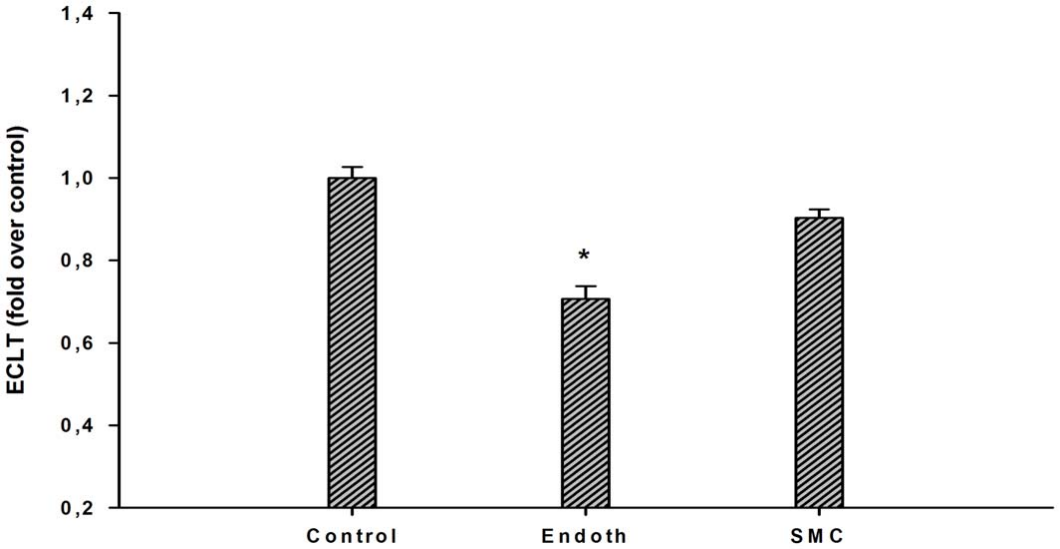
Fibrinolytic process at the surface of endothelial cells and smooth muscle cells. A. The lysis time was compared in the presence of EA.hy926 endothelial cells (endoth), smooth muscle cells (SMC) or without cells (control). Results are expressed as fold over control ratio (mean ± SEM on 6 [EC and SMC] or 4 [membrane alone] independent experiments performed in triplicate). ANOVA p<0.001, *<0.05 vs control, Dunnett’s post-hoc test.

**Figure 4:**
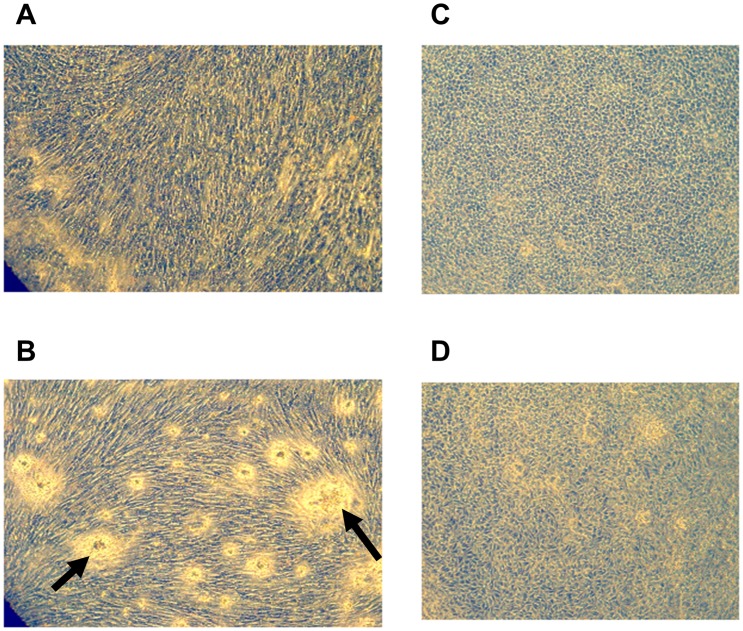
Morphology of cells. Fibrinolysis does not affect cell health. EA.hy926 endothelial cells (panels C and D) show a cobblestone morphology and smooth muscle cells (panels A and B) present a spindle shape, both of which are conserved after fibrinolysis (panels B and D). The arrows in panel B indicate fibrin residues on the surface of SMCs after fibrinolysis.

EA.hy926 endothelial cells and primary human smooth muscle cells (SMCs) were inoculated on semiporous PET membranes located inside cuvettes adapted to the lysis timer, and grown to confluence. Fig. 1 shows the equipment (cuvette, membrane, ring in metacrylate) used for cell culture and fibrinolysis recordings. An euglobulin fraction was added to the cuvette, coagulation was triggered by addition of thrombin, and fibrinolysis was allowed to proceed. The course of the lysis process is shown in Fig. 2. The lysis time calculated from the fibrinolysis curve, plotted on Figure 3, was significantly decreased (by 30%) in the presence of EA.hy926 endothelial cells, compared to a control, unseeded PET membrane. In contrast, PET membrane inoculation with SMCs did not significantly alter the lysis time (Fig. 3). To check the integrity of the culture cells, they were analyzed by optical microscopy before and after fibrinolysis. Fig. 4 shows that, despite the presence of fibrin residues after the fibrinolysis assay, EA.hy926 endothelial cells and SMCs displayed unaltered cobblestone and spindle-shaped morphology, respectively. LDH analysis confirmed that the cells were in good health (results not shown).

### Effects of TNF-α on the Fibrinolytic Process at the Surface of EA.hy926 Endothelial Cells and SMCs

**Figure 5:**
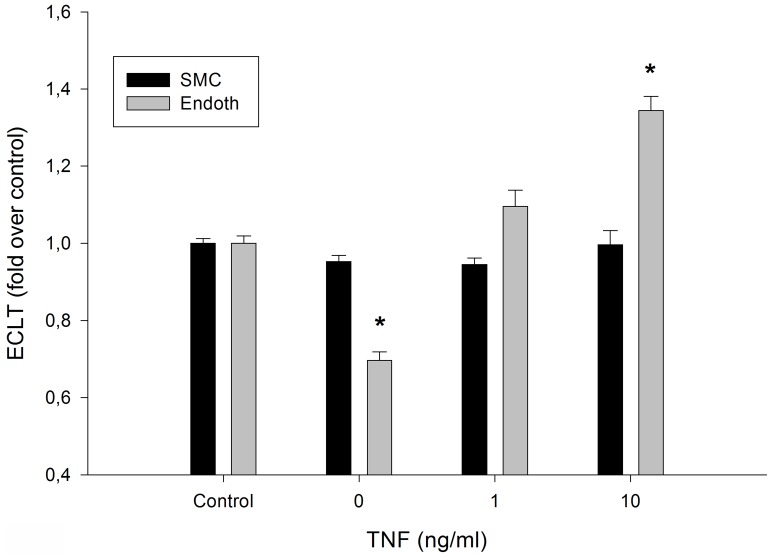
Effects of TNF-α on the fibrinolytic process at the surface of cells. The ECLT time was compared in the absence or presence of EA.hy926 endothelial cells (endoth) or smooth muscle cells (SMC) in culture medium supplemented with TNF-α at the indicated concentrations. Black histograms: SMC; gray histograms: EA.hy926 endothelial cells (endoth). Results are expressed as fold over control ratio (mean ± SEM on 4 independent experiments performed in triplicate). ANOVA <0.001, * <0.05 versus control, Dunnett’s post-hoc test.

In a second experiment, EA.hy926 endothelial cells and SMCs were exposed to tumor necrosis factor (TNF)-α for 24 hours prior to washing and loading with the euglobin fraction. TNF-α is known to have an antifibrinolytic action. Results are displayed in Fig. 5. The profibrinolytic activity of the EA.hy926 endothelial cells was confirmed. However, when EA.hy926 endothelial cells were exposed to TNF-α (1 ng/ml and 10 ng/ml), a stepwise increase in the lysis time was observed. The lack of effect of SMCs on clot lysis time was also confirmed, even when the cells were exposed to TNF-α. In an attempt to understand the differential reactivity of the two cell types to TNF-α, we measured t-PA and PAI-1 levels in the culture medium after a 24 h treatment period. Table 1 shows that, in the medium conditioned with non-exposed cells, the PAI-1/t-PA ratio was similar for both cell types. Exposure to TNF-α (10 ng/ml) significantly increased the PAI-1/t-PA ratio for both cell types, with a larger effect on SMCs.

**Table 1:** PAI-1 and t-PA in the culture medium after stimulation.

Mean ± SD	PAI-1 (ng/ml)	t-PA (ng/ml)	PAI/t-PA ratio
**Endothelial cells**			
Control	351±56	19.8±4.9	7.1±1.8
TNF (10 ng/ml)	654±225	23.9±4.3	11.7±2.8
**SMCs**			
Control	135±16	46.4±4.8	7.6±1.5
TNF (10 ng/ml)	273±38	36.5±2.7	18.2±7

TNF-α triggers an anti-fibrinolytic response in endothelial cells (ECs) and smooth muscle cells (SMCs). TNF-α was added to the culture medium of the indicated cells at a 10 ng/ml final concentration. After 24 hours, the culture supernatant was harvested and the total (active, latent and complexed) PAI-1 and t-PA protein concentrations were quantified by an ELISA test. Results were expressed as PAI-1 concentration (column 1), t-PA concentration (column 2) and PAI-1 concentration/t-PA concentration ratio (column 3) (mean ± SEM on 3 independent experiments performed in duplicate). ANOVA <0.001, * <0.05 vs control, Dunnett’s post-hoc test.

### Effect of Native LDL and Mox-LDL on the Fibrinolytic Processes at the Surface of EA.hy926 Endothelial Cells

**Figure 6:**
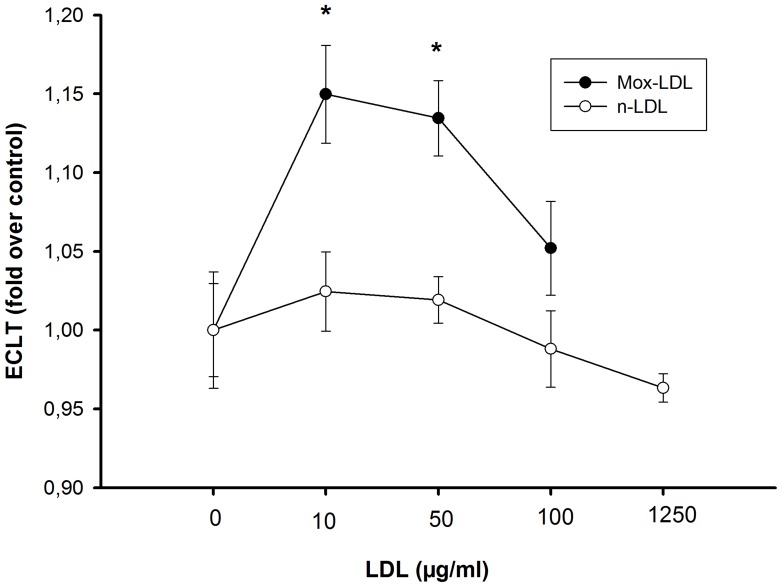
Effects of native LDL and Mox-LDL on the fibrinolytic process. Mox-LDL antagonizes the profibrinolytic effect of endothelial cells. The clot lysis time (ECLT) in the presence of EA.hy926 endothelial cells was measured after addition of native-LDL (open circles) or Mox-LDL (solid circles) at 10, 50, and 100 µg/ml final concentrations and compared to control conditions. Results are expressed as fold over control ratio (mean ± SEM on 3 Mox-LDL and 3 native LDL independent experiments performed in triplicate). ANOVA <0.001, *<0.05 vs control, Dunnett’s post-hoc test.

**Figure 7:**
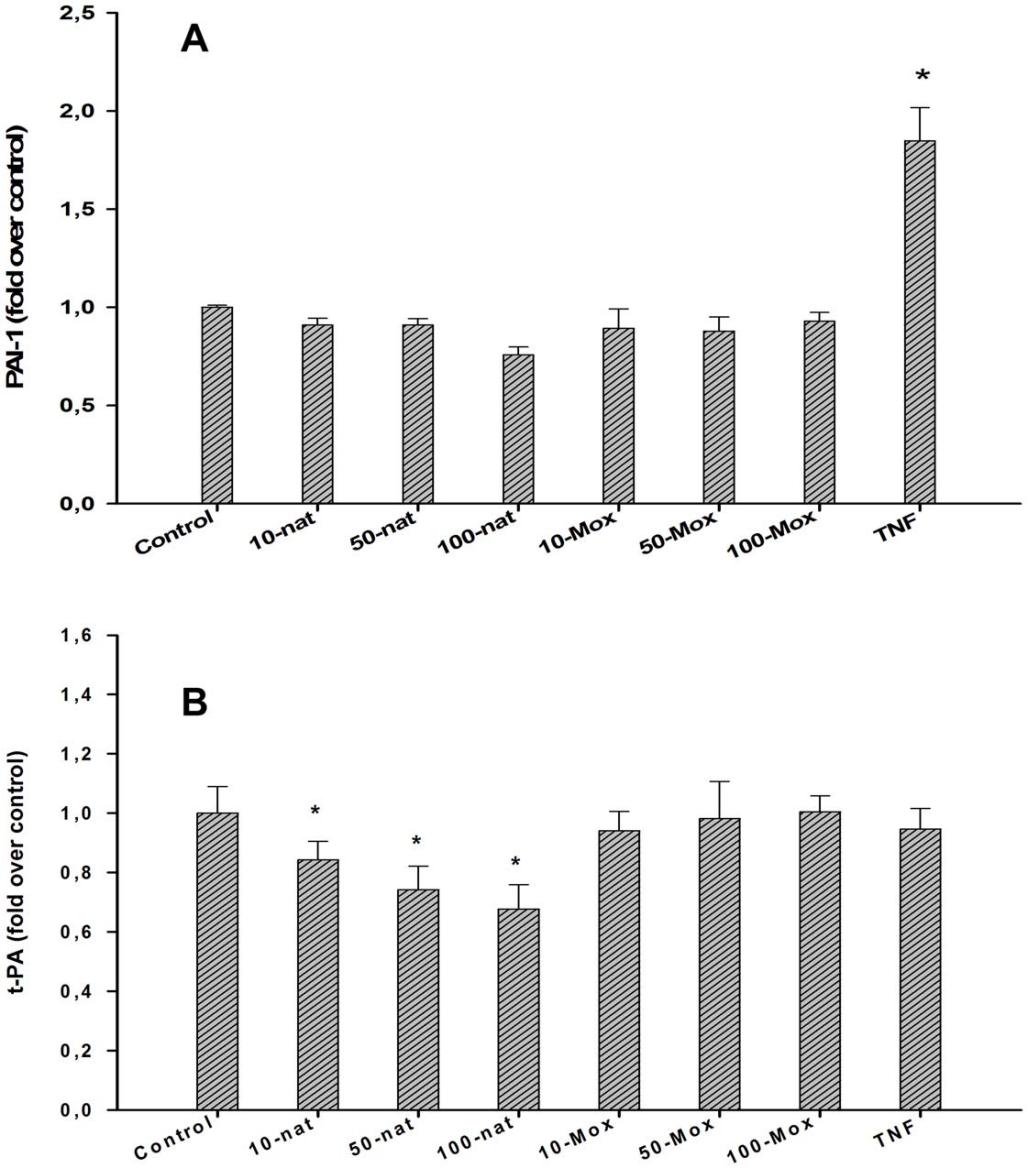
Effects of native LDL and Mox-LDL on the PAi-1 and t-PA release. MoxLDL does not interfere with PAI-1 or t-PA expression in EA.hy926 endothelial cells. Native(nat)- or Mox (Mox)-LDL were added to the endothelial cell line EA.hy926 culture medium at final concentrations of 10, 50 and 100 µg/ml and TNF-α at 10 ng/ml. After 24 h, the culture supernatant was harvested and the total (active, latent and complexed) PAI-1 and t-PA concentrations were quantified by an ELISA test. Results are expressed as fold over control of PAI-1(panel A) and t-PA (panel B) concentrations (mean ±SEM on 6 [control native and Mox-LDL] or 4 [TNF] independent experiments performed in duplicate). ANOVA <0.001, *<0.05 vs control, Dunnett’s post-hoc test.

In a third experiment, we monitored the effects of LDL and Mox-LDL exposure on the clot lysis time. There were no changes in fibrinolysis when EA.hy926 endothelial cells were exposed to native LDL (24 hours) at doses of 10, 50, 100 and up to 1250 µg/ml, Fig. 6. However, treatment of EA.hy926 endothelial cells with 10 and 50 µg/ml of Mox-LDL increased the lysis time by 15 and 13%, respectively, although this effect was decreased at higher concentrations of 100 µg/ml. A final concentration of 1250 µg/ml could not be reached in this case because of the dilution related to MPO treatment. The effects of Mox-LDL were very reproducible: Identical results were obtained during the course of three independent complete procedures involving healthy donor LDL isolation, LDL oxidation, cell culture, healthy donor euglobulin fraction preparation and fibrinolysis. Again, we monitored PAI-1 and t-PA secretion subsequent to LDL and Mox-LDL treatment and, as shown in figure 7, there was no effect (except for a decrease of t-PA expression upon LDL treatment), in contrast to what was observed with TNF-α treatment.

We, therefore, sought to investigate the effect of mox-LDL on the expression of factors that have a major influence on fibrinolysis, namely, in addition to PAI-1 and t-PA, uPAR and annexin II, the PA receptors known to be expressed by endothelial cells, and also plasminogen, α2-antiplasmin, α2-macroglobulin, FXIII, FXI, FXII, and kallikrein, which are believed to be expressed by hepatocytes, and LRP (a PAI-1/t-PA complex receptor), expressed by macrophages [20], [21]. Note that the activity of the plasminogen receptor and another t-PA receptor recently characterized [22], [23], [24] could not be tested because the genes have not yet been identified. Thus, RNA was extracted from EA.hy926 endothelial cells treated for 24 hours with LDL and Mox-LDL and analyzed by qRTPCR using appropriate primers. This experiment confirmed that plasminogen, α2-antiplasmin and α2-macroglobulin, FXIII, FXI, FXII, kallikrein and LRP are not expressed in EA.hy926 endothelial cells, even at basal levels (results not shown). There were no changes observed in mRNA levels of annexin II (a t-PA receptor [t-PAR]), LOX-1 and CL-P1 (the two scavenger receptors previously reported to be expressed by endothelial cells [25], [26]) t-PA, PAI-1 and u-PAR.and ELISA showed that there was no significant variation in the protein levels of PAI-1, t-PA and the membrane or soluble forms of uPAR either. Native LDL however slightly decreased t-PA expression.

In contrast to another pro-inflammatory stimulus, TNF-α, which acts on PAI-1 and tPA levels, Mox-LDL at patho-physiological concentrations did not interfere with the protein levels (expressed by the EA.hy926 endothelial cell line) of any known fibrinolysis factors, as recorded by ELISA.

## Discussion

Several hypotheses have been proposed to explain atheroma plaque development, two of which involve fibrin deposition and lipid accumulation as causal agents. Although involvement of the latter factor has now been extensively documented, the role of fibrin deposition has been investigated much less. To explore whether there could be a link between these two hypotheses, we adapted a device [27] (the lysis timer) so as to be able to monitor fibrinolysis in real-time at the endothelial cell surface. Our observations enable us to propose a new model integrating the two hypotheses, in which exposure of endothelial cells to Mox-LDLs, even at low concentrations, was associated with a reduced capacity to eliminate the fibrin clot, thereby enhancing endothelial permeability and foam cell accumulation.

Our data are in agreement with previous findings suggesting that the endothelial cell membrane plays a central role in the balance between coagulation and fibrinolysis. The normal endothelium is known for its capacity to inhibit coagulation and to favor fibrinolysis [28]. However, most studies performed *in vitro* have monitored fibrinolysis indirectly by quantification of endothelial cell production of t-PA and PAI-1. Rare attempts to monitor fibrinolysis itself used technically complicated methods and a system in which not all components involved in fibrinolysis were reconstituted simultaneously [29]; the results regarding the effects of endothelial cells on fibrinolysis were inconclusive. In contrast, we directly monitored pericellular fibrinolysis in real-time. We started with an euglobulin fraction containing the main factors involved in fibrin network formation and lysis, i.e., FXIII, fibrinogen, plasminogen, PAI-1 and t-PA; these factors were simultaneously put into contact with EA.hy926 endothelial cells before thrombin addition, allowing spontaneous lysis. This method allowed us to observe a profibrinolytic effect of EA.hy926 endothelial cells that was physiologically relevant because it was not observed with control SMCs.

Thus, despite a similar secreted PAI-1/t-PA ratio (with or without treatment with TNF-α, an inflammatory factor known to interfere with fibrinolysis), only EA.hy926 endothelial cells and not SMCs had an effect on fibrin clot lysis time (in agreement with Handt et al [29]). These observations suggest that factors other than the two main fibrinolysis modulators could be involved. From this point of view, the action of endothelial cells is reminiscent of the observed profibrinolytic activity of monocytes/macrophages [30], these two cell types share direct contact with blood in physiological conditions and the expression of specific receptors that are documented to enhance the fibrinolytic action of t-PA, urokinase and plasminogen [14], [24], [31]. Thus, in the presence of soluble t-PAR and t-PA bound to immobilized t-PAR, t-PA exhibited 34- and 90-fold increases in plasminogen activation, respectively. We, therefore, attempted as extensive a study as possible of genes coding for PAI-1, PA and their receptors but also other proteins involved in the control of fibrinolysis. We confirmed that genes previously reported not to be expressed in endothelial cells (plasminogen, α2-antiplasmin and α2-macroglobulin, LRP, FXIII, FXII, FXI, kallikrein were not expressed in EA.hy926 endothelial cells even under basal conditions, as their mRNA was undetectable in qRTPCR. In contrast, genes coding for annexin II, u-PAR, PAI-1 and t-PA were expressed although not regulated by Mox-LDL treatment. ELISA analyses of t-PA, PAI-1 and soluble and membrane u-PAR confirmed that there was no change at the protein level. However native LDL treatment induced a slight but significant reduction in t-PA secretion even if this did not come with any change in fibrinolysis. Therefore, all our results suggest that none of the molecules well known to regulate fibrinolysis is involved in the antifibrinolytic effect of Mox-LDL. This is in contrast to another pro-inflammatory stimulus, TNF-α, which we found exerted a similar action through different factors, namely PAI-1 and t-PA. This finding raises the question as to whether the receptor and signal transduction pathways activated by Mox-LDL and TNF-α are different.

The TNF-α receptor and signal transduction pathways have been well described [32], and as they were only used as a control in order to analyze the effects of Mox-LDL in our experiments, we believe that an extensive analysis of these pathways is beyond the scope of this paper. However, much less information is available on the oxLDL signal transduction pathways and none regarding Mox-LDL. Therefore, we attempted a preliminary dissection of the molecules involved in signal transmission after Mox-LDL treatment.

A first experiment targeted LOX-1, the scavenger receptor that has been reported in the literature to bind oxLDL and to mediate its effects [33]. The results showed that neutralizing anti-LOX-1 antibodies are not able to interfere with Mox-LDL signal transduction (as monitored by IL-8 induction) at concentrations that inhibit oxLDL signaling (results not shown). This finding suggests that Mox-LDL signal transduction is not mediated by LOX-1, which was confirmed by the lack of effect of Mox-LDL on the expression of the LOX-1 gene (results not shown) as opposed to oxLDL that has been reported to increase LOX-1 expression [34]. We, therefore, analyzed the expression of other putative receptors of the scavenger family and confirmed that most receptors of the family, namely SRB, SRD and SRF (reported to be expressed in macrophages or non endothelial cell types), are not expressed in endothelial cells. Of the two receptors (CL-P1 and LOX1) reported to be expressed in endothelial cells, only LOX-1 was expressed in the EA.hy926 cell line. It is, therefore, likely that Mox-LDL acts through an as yet unknown receptor. Finally, the use of specific inhibitors allowed us to investigate the involvement of the main pathways that have been reported to be involved in oxLDL signal transduction. Thus, we co-treated cells with Mox-LDL and either PD98059, SP600125, SB202190, GW5074, wortmanin or calphostin C, specific inhibitors of ERK 1 and 2, JNK, p38, Raf, IP3K and pKC, respectively. Although some of the inhibitors demonstrated an effect on the basal level of IL-8, none specifically interfered (neither increasing nor decreasing) with the Mox-LDL-increased IL-8 mRNA level. This finding again suggests that Mox-LDL elicits a signal transduction pathway different from that triggered by Cu-oxLDL. Finally, an intriguing observation was the effect of Mox-LDL on fibrinolysis at low but not at higher concentrations. This result is reminiscent of previous observations, for example the biphasic and contrasting effects on tPA and PAI-1 secretion of different concentrations of steroid hormones, such as 17βestradiol or progesterone [35]. Although we do not have a definite explanation for this phenomenon, it may be related to various mechanisms, including a template effect, aggregation of the LDL particles, a negative feedback loop or uptake of oxLDLs by 2 different receptors. That our Mox-LDL preparations are able to enhance IL-8 production argues against a loss of effect subsequent to aggregation. To assess the two last hypotheses would require identification of the receptor(s).

OxLDL (Mox-LDL) plays a role in cardiovascular disease. Another factor, impaired fibrinolytic function, was also proposed as a main participant a long time ago. This suggestion originated from observations by Virchow and was later supported by circumstantial observations [36], [37]. Clinical studies, always based on plasma tPA and PAI-1 levels, have reported that the risk of ischemic cardiovascular events is increased in patients with impaired plasma fibrinolytic function [38], [39], [40]. Our observations establish that physiological concentrations of Mox-LDL impairs *bona fide* fibrinolysis at the endothelial cell surface and suggest a different pathway than that involving TNF-α. This pathway and the receptor triggering it remain to be identified.

In summary, our data link the current favorite hypothesis that modified LDL has a role in atheroma plaque formation and an old suggestion that fibrin may also play a causal role. Our data suggest a means of completing the paradigm of atherosclerosis: Modified LDL enhances fibrin deposition locally (current results); fibrin deposits enhance endothelial permeability [17], [18], [19]; and this effect allows subendothelial accumulation of lipid and foam cells [41].

The mechanisms by which Mox-LDL can modify pericellular fibrinolysis will be the subject of future investigations.

## Materials and Methods

### Ethics Statement

Blood sampling was approved by the CHU Charleroi hospital ethics committee (Comité d’Ethique I.S.P.PC: OM008). The studies conform to the principles outlined in the Declaration of Helsinki. Written consent was obtained from the donors.

### Cell Culture

EA.hy926, an endothelial cell line derived from the human umbilical vein was used. This cell line results from the fusion between HUVEC (human umbilical vein endothelial cells) primary cells and a thioguanine resistant clone of A549 a pulmonary adenocarcinomic human alveolar basal epithelial cell line. EA.hy926 endothelial cells closely resemble HUVEC and retain characteristics of differentiated endothelium such as Weibel-Palade bodies, expression and or secretion of fibrinolysis and coagulation factors, expression of cell adhesion molecules as well as uptake modified LDL [42], [43], [44], [45] and uptake modified LDL [46]. Endothelial cell lines are commonly used in *in vitro* studies to avoid problems associated with the use of primary culture, because primary endothelial cells have a limited lifespan and display characteristics that differ from batch to batch as a result of their multidonor origin. Immortalized endothelial cell lines are generally better characterized and more stable in their endothelial traits than endothelial cell primocultures [47]. Cells were grown in T-75 flasks with Dulbecco’s modified Eagles’ medium (DMEM) containing 10% fetal bovine serum (FBS), 2% of a 50× concentrated HAT (1000 µM hypoxanthine, 0.4 µM aminopterin, 16 µM thymidine) solution, 1% of a 100× concentrated solution of non essential amino acids, sodium pyruvate (1 mM) and antibiotics (penicillin 100 U/ml, streptomycin 100 υg/ml), all from Lonza, Verviers, Belgium. Primary human aortic Smooth Muscle Cells (AoSMC, Lonza) were grown in T-75 flasks with Smooth Muscle Cell Growth Medium-2 Buletkit® (SMGM-2, Lonza).

### Assessment of Pericellular Fibrinolysis

A fully computerized semi-automatic 8-channel device was designed to measure fibrin formation and degradation in adapted circular microcuvettes [27]. The method has been fully described previously [27]. In brief, 40000 cells (EA.hy926 endothelial cells or SMC) were inoculated on a polyethylene terephthalate (PET, Whatman SA, Louvain-la-Neuve, Belgium) microporous membrane (coated overnight with Type I collagen, 0.1%, Roche), in glass circular micro-cuvettes (51 mm^2^, Fig. 1) and grown for 5 days until confluence. The cells were incubated for 24 h in DMEM medium containing the studied molecules. The medium was discarded and the cells were washed three times with HBSS before fibrinolytic tests. Euglobulin fractions (EF) were prepared from a frozen plasma pool from 5 volunteers. Three hundred microliters of acetic acid (0.25%) and 3.6 ml of desionized water were added to 400 µl of plasma (final pH ≅ 5.2). The samples were put into melting ice for 20 min and centrifuged at 4000 g for 10 min at 4°C. The supernatant was discarded and the pellet was resuspended in 400 µl of HBSS (HEPES 25 mM, pH: 7.3). This fraction contains fibrinogen, PAI-1, t-PA, plasminogen, and to a lesser extent alpha 2-antiplasmin and factor VIII [48]. Two hundred and fifty microliters of EF were added in cell-seeded cuvettes. The microcuvettes were inserted in the apparatus at 37°C. Clot formation was started by the addition of 50 µl of thrombin (Hyphen-Biomed, reconstituted with RPMI 1640, 25 mM HEPES, 1.5 U/ml). Coagulation and fibrinolysis were then allowed to proceed. The Euglobulin Clot Lysis Time (ECLT) expressed in minutes (range: from 5 to 9999) was determined from a mathematical analysis of the recorded lysis curve. As the ECLT results in part from the balance between t-PA and PAI-1 activities, recombinant human TNF-α (1 and 10 ng/ml) (Sigma, St Louis, MO) was used to verify the reactivity of cells because it is known to increase the ratio of PAI-1/t-PA secreted by the endothelium. The cytotoxicity of the process was assessed by measuring the lactate dehydrogenase (LDH) activity in the cell supernatant and was always found to be <10% of the cellular content.

### Computer Fibrinolysis Analysis

The course of the lysis process is shown in Fig. 2. The x- and y-axis represent, respectively, the time and evolution of the signal recorded by the device. At the end of fibrinolysis, the curve is analyzed with a mathematical algorithm. The first and second derivatives are computed by convolution matrix. These calculations determine the peak time to clot lysis (B point, first derivative), and the end of the complete fibrinolysis process (C point, when the first and the second derivatives are at the background level ≅0). In our experiments, the lysis time was determined by the C point and expressed in minutes.

### Protein Analysis

The PAI-1, t-PA and u-PAR protein concentrations in the medium were quantified using ELISA tests (Zymutest PAI-1 Antigen, Zymutest t-PA Antigen, u-PAR antigen Hyphen-Biomed, France), which detect active and inactive (latent) forms of PAI-1 and t-PA, as well as t-PA/PAI-1 complexes or u-PAR respectively. Raw data are presented as supernatant concentrations. Results are also expressed as the ratio of PAI-1 antigen to t-PA antigen (PAI-1/t-PA ratio).

### Recombinant MPO Preparation

Recombinant MPO was prepared as described previously [49]. Each batch solution was characterized by its activity (U/ml), protein concentration (mg/ml) and specific activity. Peroxidative activity was determined using *o*-dianiside as the substrate. Protein concentration was measured using the Lowry assay, with ovalbumin as a standard. Each batch was checked for endotoxin using the LONZA Kit. Concentration was always less than 100 pg/ml, which, taking into account the final dilution of the MPO-treated LDL fraction, would contribute a final concentration of less than 0.1 pg/ml to the Mox-LDL supplemented medium added to the cells. This concentration is 5000× less than the amount shown not to have any effect on IL-8 production by EA.hy926 cells.

### Isolation of LDL and Mox-LDL Preparation

Both lipoprotein particles were isolated from plasma from sterile blood pouches using density-gradient ultracentrifugation. The LDL fraction (d = 1.019–1.063) was stored under nitrogen at 4°C in the dark and oxidized according to the procedure described below: Prior to oxidation, LDL were gel filtered (PD-10 column, Pharmacia) and 1.6 mg of LDL were oxidized by 2.1 chlorinating units of recombinant MPO, to form the oxidized LDL (Mox-LDL) in the presence of 1 mM H_2_O_2_ in 2 ml PBS at pH 6.5 for 5 minutes [50]. LDLs were desalted again after MPO treatment. Protein concentration was measured by the Lowry assay, using ovalbumin as a standard.

### Statistics

SigmaStat® software (SPSS, 3.0) was used for the analysis. Data are presented as mean ± SEM and were evaluated by one-way ANOVA, with Dunnett’s *post-hoc* test.
